# Data on the productivity of plant cover of the main types of soils of the North-Western precaspian in connection with the dynamics of ecological factors

**DOI:** 10.1016/j.dib.2019.103713

**Published:** 2019-03-15

**Authors:** Gasan Gasanov, Tatiana Asvarova, Kamil Hajiyev, Rashid Bashirov, Aishat Abdulaeva, Zaira Akhmedova, Shamil Salikhov, Nurjagan Ramazanova, Viktoriya Semenova, Radjab Usmanov, Aytemir Aytemirov, Magomed Musaev, Nurulislan Magomedov

**Affiliations:** a Precaspian Institute of Biological Resources of Dagestan Scientific Center of the Russian Academy of Sciences, St. M. Hajiyev, 45, Makhachkala, 367000, Russia; b Daghestan State University, 21, Dahadaeva Street, Makhachkala, 367025, Russia; c Federal State Budgetary Educational Institution of Higher Education, “Dagestan State Agricultural University Named After M.M. Dzhambulatov”, 367032, The Republic of Dagestan, Makhachkala, M. Gadzhieva Street, 180, Russia; d Federal State Budgetary Scientific Institution Kisriev Dagestan Research Institute of Agriculture, Akushinskogo Avenue, Makhachkala, The Republic of Dagestan, 367014, Russia

**Keywords:** Productivity of phytomass, Light-chestnut soil, Meadow-chestnut soil, Hydrothermal conditions, Salt-forming ions, Salinity, Species composition phytocenosis, Aboveground and underground phytomass, Coefficient the use of the FAR, Aridity integral, Integral moisture

## Abstract

The data of the researches describes the were to establish the species composition of pasture cenoses and the productivity potential of light-chestnut and meadow-chestnut soils under different climatic conditions and in different periods of the year in the Terek-Kuma lowland of the North-Western Precaspian. Two peaks of productivity of phytocoenosis have been observed: the first is – ephemeral synusia in the middle of May- early June; second – motley grass and saltworts in the second half of September. The data on receipt of photosynthetically active radiation (PAR) on the soil surface and the coefficient of its use over the years and periods of the year depending on the hydrothermal conditions and dynamics of harmful salts in the soil are given. On light-chestnut soil formed cereals-wormwood, grass-cereals, wormwood-ephemeral in combination with wormwood-saltworts association, and meadow-chestnut soil – ephemera-wormwood. The phytocenosis on light-chestnut soil is inherent in the maximum species diversity – 35 species. On meadow-chestnut soil there are only 25 species. The items of the changes in the species composition of phytocenoses depending on the environmental factors are considered. The data in this article support and augment information presented in the research articles [1], [2], [3], [4], [5], [6], [7].

**Table:** Specifications table

N	*Biology*
More specific subject area	*Productivity of phytomass*
Type of data	*Tables, figures, text file.*
How data was acquired	*Calculate the coefficient of use of the PAR was performed, using the formula A. Nichiporovich to determine the theoretically possible yield of plants. Climatograms over the years are compiled according to the method of Walter. The stocks of aboveground and underground plant material was taken into account by the method of A. Titlyanova. The names of plants are given by S. Cherepanov.*
Data format	*Raw, descriptive and inferential*
Experimental factors	*The area of the experimental plots of 100 m*^*2*^*, surrounded by an iron grid to avoid the loss of phytomass by cattle. Each of the sites is divided into 100 permanent sites, an area of 1 m*^*2*^*, polyethylene twine. Sites outside the protected conditions were taken as a background.*
Experimental features	*Studies on biological productivity and species composition of plant communities were conducted in 2011-2018 on light-chestnut and meadow-chestnut soils of Kochubey biosphere station (KBS) of PIBR DNC RAS.*
Data source location	*The geographical coordinates of the experimental sites were determined using GPS-Navigator, which corresponded to the light chestnut and meadow-chestnut soils – Google Earth Pro 44.40848 46.24652*
Data accessibility	https://data.mendeley.com/datasets/s438x9m62x/edit#file-0b7f964e-caf6-4a54-b456-9050dcd43f72 https://data.mendeley.com/datasets/s438x9m62x/edit#file-3eff1aea-96b2-4b58-9718-70a0997b0907
Related research article

**Table:** 

**Value of the data** • The datasets were obtained during the years 2011–2018 in the pasture areas of the North-Western Precaspian region in semi-desert conditions. This is an important region of livestock and crop production for Russia. • The established patterns of formation of aboveground and underground phytomass is the basis for the rational management of production processes in natural and artificially modified ecosystems, for improving soil fertility and landscape productivity. • The data can serve as a benchmark for future research on the formation of the species composition and productivity of phytomass not only in the North-Western Precaspian under the action of various environmental factors but also in other semi-arid regions. • These data can be used for the organization of sustainable pasture systems when allocating land for haymaking. • These data can be useful in a statistical approach to analysis, in calculating the integral of moisture, integral of aridity, coefficient the use of photosynthetically active radiation (PAR), regression equations and various mathematical dependencies.

## 1 Data

This article includes descriptive data and statistical data (95% confidence intervals) on the impact of environmental factors on the productivity of light chestnut and meadow chestnut soils during the years 2011–2018. The dynamics of the accumulation of live aboveground biomass, the PAR use coefficient grazing plant communities’ phytocenoses and integrals of moisture and aridity of climate over periods of a year. Dynamics of environmental factors and the content of harmful salts in soil horizons are shown. This is an important long-term study at the Kochubey biosphere station on the territory of the Terek-Kuma lowland of the North-Western Precaspian to ensure the collection of data on soil and plant cover, crop yield and its dependence of climatic factors in different years.

## 2 Experimental design, materials, and methods

Studies on biological productivity and species composition of plant communities were conducted in the years 2011–2018 on light-chestnut and meadow-chestnut soils of Kochubey biosphere station (KBS) of PIBR DNC RAS. The main physical and chemical parameters of these soils are: light chestnut-soil density 0–23 cm 1.18 g/cm^3^, the lowest moisture capacity (FC) – 18.8%, humus content of 1.1%, P_2_O_5_ – 0.53 mg/100 g, K_2_O – 30.8 mg/100 g, meadow chestnut-respectively 1.18 g/cm^3^, 25.6%; 1 %, P_2_O_5_ – 0.84 mg/100 g, K_2_O – 33.8 mg/100 g.

Type of salinity in the soil horizons varies from sulfate-chloride, chloride-sulfate, salinity from weak in the upper layers to a strong downward.

**Fig. 1:**
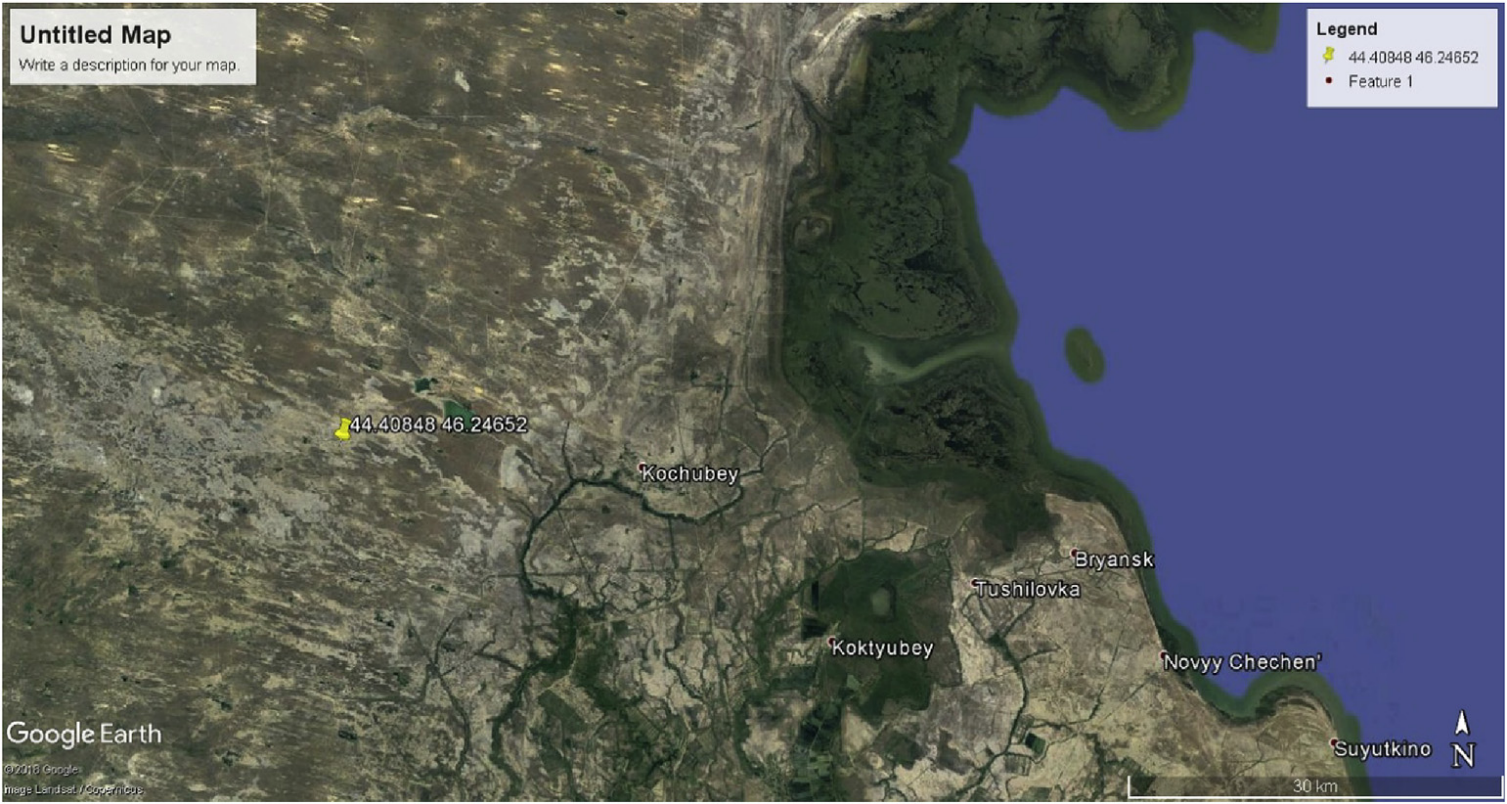
The geographical coordinates of the experimental sites were determined using GPS-Navigator, which corresponded to the light chestnut and meadow-chestnut soils – Google Earth Pro 44.40848 46.24652.

The area of the experimental plots of 100 m^2^, surrounded by an iron grid to avoid the loss of phytomass by cattle. Each of the sites is divided into 100 permanent sites, an area of 1 m^2^, polyethylene twine. Sites outside the protected conditions were taken as a background. The geographical coordinates of the experimental sites were determined using GPS-Navigator, which corresponded to the light chestnut and meadow-chestnut soils – Google Earth Pro 44.40848 46.24652 (Fig. 1).

In the assessment of phytocenotic diversity of the communities we used the principle of allocation of plant communities according to the community of biotope species composition and dominance of species.

The PAR use coefficient was calculated by the formula of A. Nichiporovich [8] to determine the theoretically possible yield of plants:

(1) $$\Upsilon =R\times 10^{8}\times K/10^{2}\times 4\times 10^{3}\times 10^{2}$$

where Y – biological yield of absolutely dry aboveground mass, kg/ha;

Rх10^8^ – number of PAR coming on 1 ha over the growing period of plants, kcal;
K – the planned coefficient of use of the PAR, %;
4х10^3^ – the amount of energy released by burning 1 kg of dry matter of biomass, kcal/kg;
10^2^ – translation kg per c of product.

To calculate the utilization of the PAR, the formula is:

(2) $$Κ=\Upsilon \times 10^{2}\times 4\times 10^{3}\times 10^{2}/R\times 10^{8}$$

In the calculations the duration of the vegetative period of plants was calculated based on the date of transition of daily average temperature of air through ±5 °C. The flow of PAR on the 1 cm^2^ soil per year in the lowlands is 50.87 kcal (213.23 Kj), including by month (kcal): January – 0.59, February – 1.99, March – 3.82, April – 5.97, May – 7.27, June – 8.48, July – 7.84, August – 6.22, September – 4.59, October – 2.57, November – 1.19, December – 0.34 [1].

Climatograms over the years are compiled according to the method of Walter [9], in which, during dry periods the curve of air temperature is above the precipitation curve in the wet, on the contrary, the precipitation curve is above the curve of temperatures. To calculate the area of the plots between the lines of mean monthly air temperatures (°C) and sum of monthly precipitation (mm) used integrals: $\overset{b}{\underset{a}{\int }}\max\left(T\left(t\right)-W\left(t\right)\text{,}\;0\right)\mathrm{d}t$ – to calculate the area of dry, $\overset{b}{\underset{a}{\int }}\max\left(W\left(t\right)-T\left(t\right)\text{,}\;0\right)\mathrm{d}t$ – to moist periods.

The stocks of aboveground and underground plant material were taken into account by the method of A. A. Titlyanova [10]. Aboveground mass was determined by the method of hay, with the release fractions of living phytomass (air-dry), rags (dead parts of plants, preserved communication with the plants, the steppe felt (dead plant remains on the soil surface, deprived of communication with plants). Underground mass was determined at the same time on the same experimental plots (after cutting above-ground mass) to a depth of 60 cm by the method of the monolith. The size of the monoliths 10 × 10 × 10 cm, repeated 4 times. The names of plants are given by S.K. Cherepanov [11].

Pastures of the Terek-Kuma lowland include communities of different nature, which differ depending on the conditions of mesorelief, projective cover, height of grass stand and species diversity. A significant part of the productivity of semi-desert communities is provided by dominant species, which are grouped by life forms (Table 1).

**Table 1:** Characteristic of plant communities in terms of conservation and the natural regime of the Terek-Kuma lowland 2011–2018.

Soil	Light-chestnut	Meadow-chestnut
Plant community	Ephemeroid-wormwood- cereal	Grass-wormwood
Edifiers, dominants, and other types of grass cover plants	*Poa bulbosa, Bromus squarrosus*, *Anisantha tectorum, Agropyron desertorum*, *Eragrostic minor, Eremopyrom triticeum*, *Eremopyrom orientale*, *Artemisia lercheana*, *Artemisia taurica*, *Alyssum desertorum*, *Salsola iberica*, *Salsola australis*, *Xanthium spinosum*, *Herniaria incana*, *Silene conica*, *Ceratocarpus arenarius*	*Artemisia lercheana*, *Artemisia taurica*, *Silene conica*, *Ceratocarpus arenarius Herniaria incana*, *Alyssum desertorum*, *Poa bulbosa, Bromus squarrosus*, *Anisantha tectorum*, *Eragrostic minor, Eremopyrom triticeum*, *Salsola iberica*
**Conservation regime**		
Number of views per 1 m^2^	14–20	8–13
Height of grass, cm	30–60	20–45
Projective cover, %	70–80	40–60
Plant groups: cereals, %	50–60	10–20
Wormwood, %	15–30	30–50
Solyanka, %	5–8	5–7
Herbs, %	10–15	5–10
Half-shrubs, %	1–7	1–5
**Natural regime**		
Number of views per 1 m^2^	7–8	5–7
Height of grass, cm	10–20	10–25
Projective cover, %	30–50	30–60
Plant groups: cereals, %	15–25	10–20
Wormwood, %	30–50	30–60
Solyanka, %	5–7	5–6
Herbs, %	5–8	4–6
Half-shrubs, %	1–5	1–4

The plant cover of light-chestnut soil is represented by an ephemeroid-wormwood- cereal community, with species dominants - *Poa bulbosa, Bromus squarrosus*, *Anisantha tectorum*, *Agropyron desertorum*, *Eragrostic minor, Eremopyrom triticeum*, *Eremopyrom orientale*, *Artemisia lercheana*, *Artemisia taurica*, *Alyssum desertorum*, *Salsola iberica*, *Salsola australis*, *Xanthium spinosum*, *Herniaria incana*, *Silene conica*, *Ceratocarpus arenarius.* On meadow-chestnut soils-edificatory role of the grass-wormwood community: half-shrubs with high duration and stability in semi-desert communities *– Artemisia lercheana*, *Artemisia taurica* и другие доминирую♯ие ϑиды – *Silene conica*, *Ceratocarpus arenarius, Herniaria incana*, *Alyssum desertorum*, *Poa bulbosa, Bromus squarrosus*, *Anisantha tectorum*, *Eragrostic minor, Eremopyrom triticeum*, *Salsola iberica.*

**Fig. 2:**
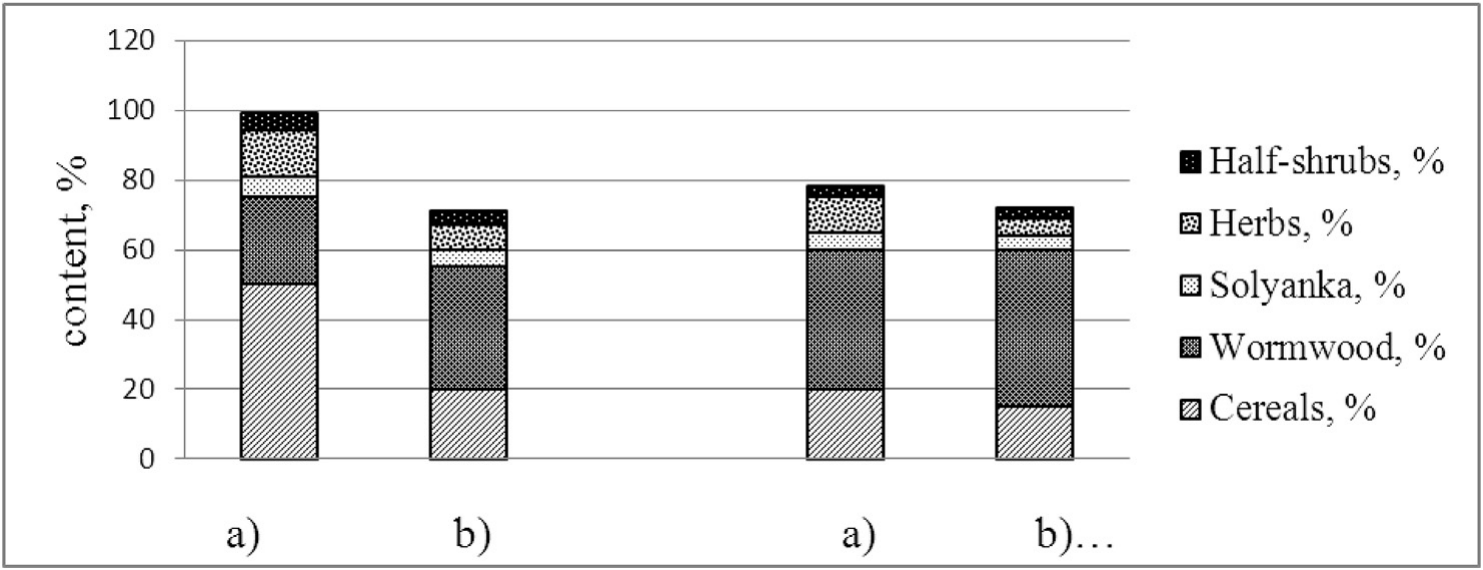
Content of life forms in plant communities of light chestnut and meadow chestnut soils of KBS for 2015–2018 years: a- reserved regime; b-natural regime.

In Fig. 2 shows a comparative characteristic of the average indicators of the share of life forms in the ephemeroid-wormwood-cereal and grass-wormwood communities with a – reserved; b– natural mode during 2011–2018 years.

The study of the life forms of the conservation regime of the ephemeroid-wormwood- cereal community showed the prevalence of grasses, ranging from 50 to 60%, low content was observed in forbs and half-shrubs on average 10% (Table 1).

The abundance of *Artemisia lercheana* and *Artemisia taurica* species prevails in the herbage with a natural regime, their content increases up to 60% in some years, and the dominant changes occur.

A comparison of modern conditions with the conservation regime has shown that the content of large-grain cereals has decreased. Data on the maximum reserves of green mass of protected conditions is 3.5 times higher than natural ones.

With a change in the moisture regime and heat supply, the amount of phytomass reserves in the protected mode (2015–2018) changes, leading to a decrease in the mortmass, i.e. rags and felt (Fig. 2).

**Fig. 3:**
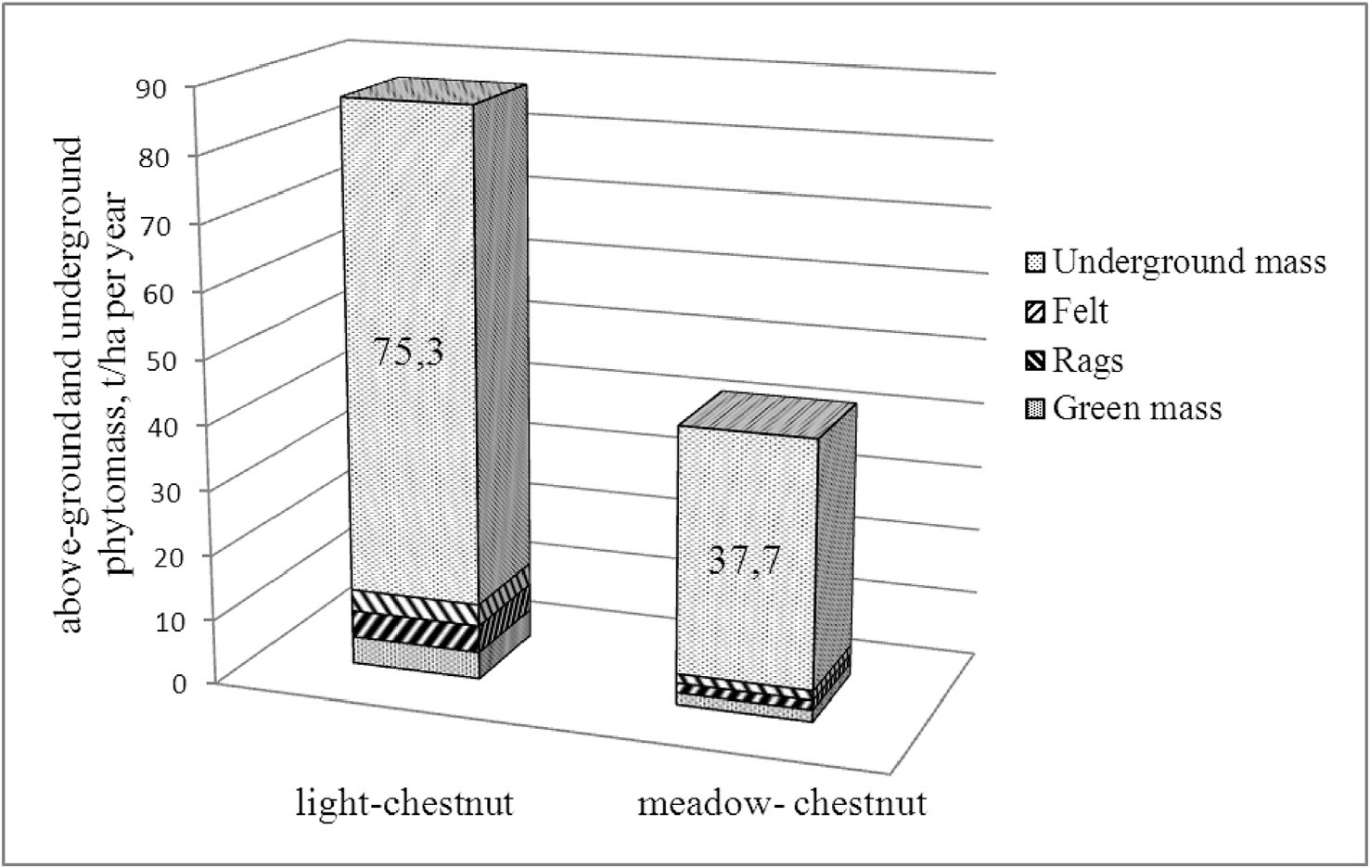
Productivity of above-ground and underground mass of plant matter of light- chestnut and meadow-chestnut soil (2015–2018), t/ha per year.

## References

[1] Gasanov G.N., Musaev M.R., Abdurakhmanov G.M., Kurbanov S.A., Adzhiev A.M. (2004). Phytomelioration of Saline Soils of the Western Caspian.

[2] Asvarova A.T., Zalibekov Z.G., Abdullayeva A.S. (2013). Impact of desertification processes on the intensity of migration radionuclides in soils of the Terek-Kuma lowland. Arid Ecosyst..

[3] Gasanov G.N., Asvarova T.A., Hajiyev K.M., Akhmedova Z.N., Abdulaeva A.S., Bashirov R.R., Sultanakhmetov S.M., Salikhov S.A. (2014). Hydrothermal conditions species composition and productivity of phytocenose in the Terek-Kuma lowland. Arid Ecosyst..

[4] Asvarova Tatiana, Gasanov Gasan, Abdulayeva Aishat, Salikhov Shamil, Hajiyev Kamil, Bashirov Rashid, Akhmedova Zaira, Gimbatova Kabirat, Aytemirov Aytemir, Magomedov Nurulislam, Musaev Magomed (2017). Rate of migration of natural radionuclides in soil-plant cover of the north-western Precaspian region. J. Ponte.

[5] Gasanov Gasan, Salikhov Shamil, Ramazanova Nurjagan, Asvarova Tatiana, Hajiyev Kamil, Bashirov Rashid, Abdulaeva Aishat, Akhmedova Zaira, Gimbatova Kabirat, Shaikhalova Zhamilat, Semenova Viktoriya, Usmanov Radjab, Mallaliev Maxim, Aytemirov Aytemir, Musaev Magomed, Magomedov Nurulislan (2017). Floristic composition and productivity of mountain pastures of Dagestan. J. Ponte.

[6] Gasanov G.N., Asvarova T.A., M Hajiyev K., Bashirov R.R., Abdulayeva A.S., Akhmedova Z.N., Salikhov ShK., Ramazanova N.I., Usmanov R.Z., Aytemirov A.A., Magomedov N.R., Musaev M.R. (2018). The productivity of meadow-chestnut soils of the north-western Precaspian region according to the dynamics of the environmental factors. Int. Agric.J..

[7] Gasanov Gasan, Asvarova Tatiana, Hajiyev Kamil, Bashirov Rashid, Abdulayeva Aishat, Akhmedova Zaira, Salikhov Shamil, Ramazanova Nurjagan, Semenova Viktoria, Usmanov Radjab, Aytemirov Aytemir, Magomedov Nurulislam, Musaev Magomed (2018). The potential productivity of light-chestnut soils of the North-Western Precaspian region in connection with the dynamics of environmental factors. Open Access Libr J. (OALib Journal).

[8] Nlchiporovich A.A. (1963). On ways of improving the productivity of photosynthesis in crops. Photosynth. Prod. Plants.

[9] Walter N.D. (1964). Vegetation der Erde in oko-physiolohischeiBetrachtung, Die tropicshen und subtropischen Zonen, Vienna.

[10] Titlyanova A.A. (1988). Productivity of Grass Ecosystems, Biological Productivity of Grassland Ecosystems, Geographical Patterns and Ecological Characteristics.

[11] Cherepanov S.K. (1981). Vascular Plants of the USSR.

